# Supplementary material for: Single-chain tandem macrocyclic peptides as a scaffold for growth factor and cytokine mimetics
Source: Commun Biol. 2022 Jan 14;5:56. doi: 10.1038/s42003-022-03015-6 (PMC8760323; doi:10.1038/s42003-022-03015-6)
Supplement: Supplementary file 2 — Supplementary information [file 42003_2022_3015_MOESM2_ESM.pdf]

Supplementary Information

Single-chain tandem macrocyclic peptides as a scaffold for growth factor and cytokine mimetics

Kenichiro Ito*, Yoshihiko Matsuda, Ayako Mine, Natsuki Shikida, Kazutoshi Takahashi, Kyohei Miyairi, Kazutaka Shimbo, Yoshimi Kikuchi, Atsushi Konishi

Research Institute for Bioscience Products & Fine Chemicals, Ajinomoto Co., Inc.,
1-1, Suzuki-Cho, Kawasaki-ku, Kawasaki-shi, Kanagawa, 210-8681, Japan

*Correspondence author: kenichiro.ito.qf8@asv.ajinomoto.com

Supplementary Table 1: Kinetic parameters of STaMPtides and monomeric macrocyclic peptides. The mean values and standard deviation from the results of triplicated experiments are shown.

Ligand	Analyte	k_a (s^{-1})	k_d ($M^{-1}s^{-1}$)	K_D (M)
Met-Fc	aMD4dY (Monomer)	9.8×10^5 ($\pm 6.6 \times 10^4$)	4.5×10^{-2} (4.8×10^{-3})	4.5×10^{-8} ($\pm 7.4 \times 10^{-9}$)
	aMD4dY-PA22 (STaMPtide)	4.5×10^5 ($\pm 7.6 \times 10^3$)	4.6×10^{-4} ($\pm 1.2 \times 10^{-4}$)	1.0×10^{-9} ($\pm 2.6 \times 10^{-10}$)
EPOR-Fc	EMP35 (Monomer)	6.3×10^4 ($\pm 5.3 \times 10^4$)	$<1.0 \times 10^{-5}$*	$<5.0 \times 10^{-10}$* ($\pm 6.7 \times 10^{-10}$)
	EMP-PA8 (STaMPtide)	3.4×10^5 ($\pm 4.7 \times 10^4$)	$<1.0 \times 10^{-5}$*	$<3.0 \times 10^{-11}$* ($\pm 4.0 \times 10^{-12}$)
	EMP-PA22 (STaMPtide)	7.8×10^5 ($\pm 1.6 \times 10^5$)	$<1.0 \times 10^{-5}$*	$<1.3 \times 10^{-11}$* ($\pm 2.5 \times 10^{-12}$)

*Out of the measurement range

Supplementary Table 2: Amino acid sequences of the peptides used in the study.

Construct	Sequence
aMD4dY	CRQFNRRTHEVWNLDC-OH
aMD4dY-PA8	AETCRQFNRRTHEVWNLDCGAAPAAPAPGCRQFNRRTHEVWNLDC
aMD4dY-PA22	AETCRQFNRRTHEVWNLDCGAAPAAPAPAAPAAPAPAAPAAPGCRQFNRRTHEVWNLDC
aMD4dY-PA49	AETCRQFNRRTHEVWNLDCGAAPAAPAPAAPAAPAPAAPAAPAPAAPAAPAPAAPAAPAPAAPAAPAPAGC RQFNRRTHEVWNLDC
aMD4dY-PA100	AETCRQFNRRTHEVWNLDCGAAPAAPAPAAPAAPAPAAPAAPAPAAPAAPAPAAPAAPAPAAPAAPAPAAP AAPAPAAPAAPAPAAPAAPAPAAPAAPAPAAPAAPAPAAPAAPAPAAPAAPAPAAPAGCRQFNRRTHEVWNLDC
aMD4dY-PA200	AETCRQFNRRTHEVWNLDCGAAPAAPAPAAPAAPAPAAPAAPAPAAPAAPAPAAPAAPAPAAPAAPAPAAP AAPAPAAPAAPAPAAPAAPAPAAPAAPAPAAPAAPAPAAPAAPAPAAPAAPAPAAPAAPAPAAPAAPAPAAPA APAPAAPAAPAPAAPAAPAPAAPAAPAPAAPAAPAPAAPAAPAPAAPAAPAPAAPAAPAPAAPAAPAPAAPAA PAPGCRQFNRRTHEVWNLDC
aMD4dY-GS15	AETCRQFNRRTHEVWNLDCGGGGGSGGGGSGGGGSGCRQFNRRTHEVWNLDC
aMD4dY-GS22	AETCRQFNRRTHEVWNLDCGGGGGSGGGGSGGGGSGGGGSGGGCRQFNRRTHEVWNLDC
aMD4dY-Trimer	AETCRQFNRRTHEVWNLDCGAAPAAPAPAAPAAPAPAAPAAPGCRQFNRRTHEVWNLDCGAAPAAPAPA APAAPAPAAPAAPGCRQFNRRTHEVWNLDC
EMP35	GGLYACHMGPMTWVCQPLRG
EMP-PA8	AETGGLYACHMGPMTWVCQPLRGAAPAAPAPGGLYACHMGPMTWVCQPLRG
EMP-PA22	AETGGLYACHMGPMTWVCQPLRGAAPAAPAPAAPAAPAPAAPAAPGGLYACHMGPMTWVCQPLRG
TMP-PA8	GGCADGPTLREWISFCGGAAPAAPAPGGCADGPTLREWISFCGG
EMP1	TYSCHFGPLTWVCKPQ
Peginesatide core	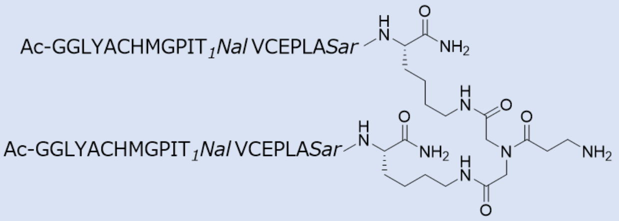

Supplementary Table 3: Approximate secretion levels of STaMPtides.

Construct	Secretion level (mg/L)
aMD4dY-PA22	110
EMP-PA8	110
EMP-PA22	73
TMP-PA8	78

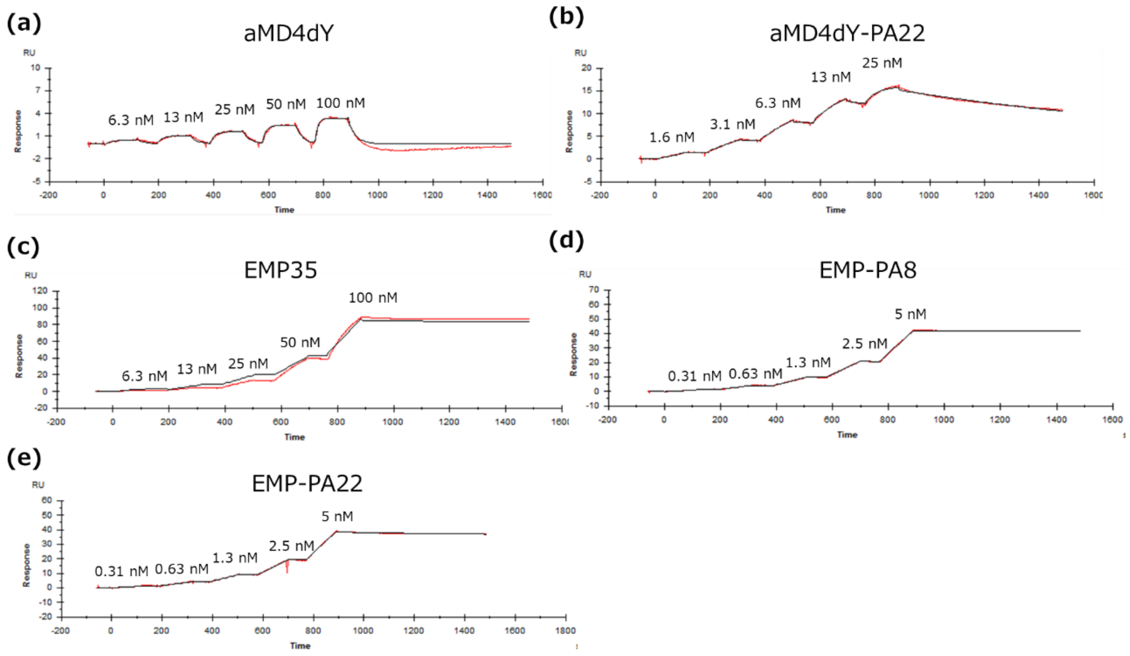

Supplementary Figure 1: SPR sensorgrams of (a) monomeric aMD4dY and (b) aMD4dY-PA22 against recombinant human Met ectodomain-Fc and (c) monomeric EMP35, (d) EMP-PA8, and (e) EMP-PA22 against rhEPOR ectodomain-Fc analyzed by Biacore T200. Measured (red) and fitted (black) curves are shown. Kinetic parameters calculated from fitted curves are listed in **Supplementary Table 1**.

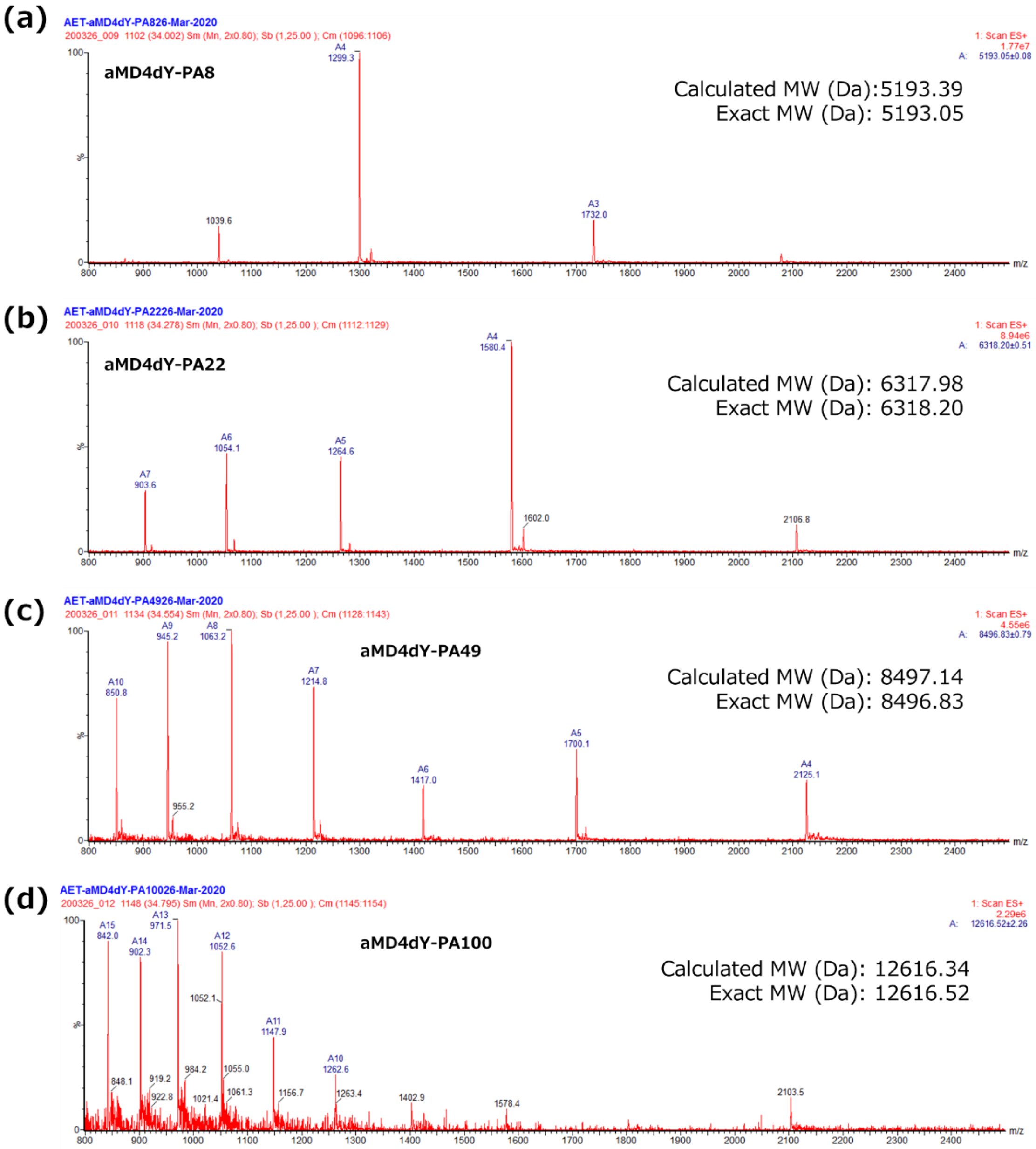
(a)
AET-aMD4dY-PA826-Mar-2020
200326_009 1102 (34.002) Sm (Mn, 2x0.80); Sb (1,25.00); Cm (1096:1106)
1: Scan ES+
1.77e7
A: 5193.05±0.08
aMD4dY-PA8
Calculated MW (Da):5193.39
Exact MW (Da): 5193.05
A4
1299.3
1039.6
A3
1732.0
m/z
(b)
AET-aMD4dY-PA2226-Mar-2020
200326_010 1118 (34.278) Sm (Mn, 2x0.80); Sb (1,25.00); Cm (1112:1129)
1: Scan ES+
8.94e6
A: 6318.20±0.51
aMD4dY-PA22
Calculated MW (Da): 6317.98
Exact MW (Da): 6318.20
A4
1580.4
A7
903.6
A6
1054.1
A5
1264.6
1602.0
2106.8
m/z
(c)
AET-aMD4dY-PA4926-Mar-2020
200326_011 1134 (34.554) Sm (Mn, 2x0.80); Sb (1,25.00); Cm (1128:1143)
1: Scan ES+
4.55e6
A: 8496.83±0.79
aMD4dY-PA49
Calculated MW (Da): 8497.14
Exact MW (Da): 8496.83
A10
850.8
A9
945.2
A8
1063.2
955.2
A7
1214.8
A6
1417.0
A5
1700.1
A4
2125.1
m/z
(d)
AET-aMD4dY-PA10026-Mar-2020
200326_012 1148 (34.795) Sm (Mn, 2x0.80); Sb (1,25.00); Cm (1145:1154)
1: Scan ES+
2.29e6
A: 12616.52±2.26
aMD4dY-PA100
Calculated MW (Da): 12616.34
Exact MW (Da): 12616.52
A15
842.0
A14
902.3
A13
971.5
A12
1052.6
1052.1
A11
1147.9
A10
1262.6
848.1
919.2
922.8
984.2
1021.4
1055.0
1061.3
1156.7
1263.4
1402.9
1578.4
2103.5
m/z

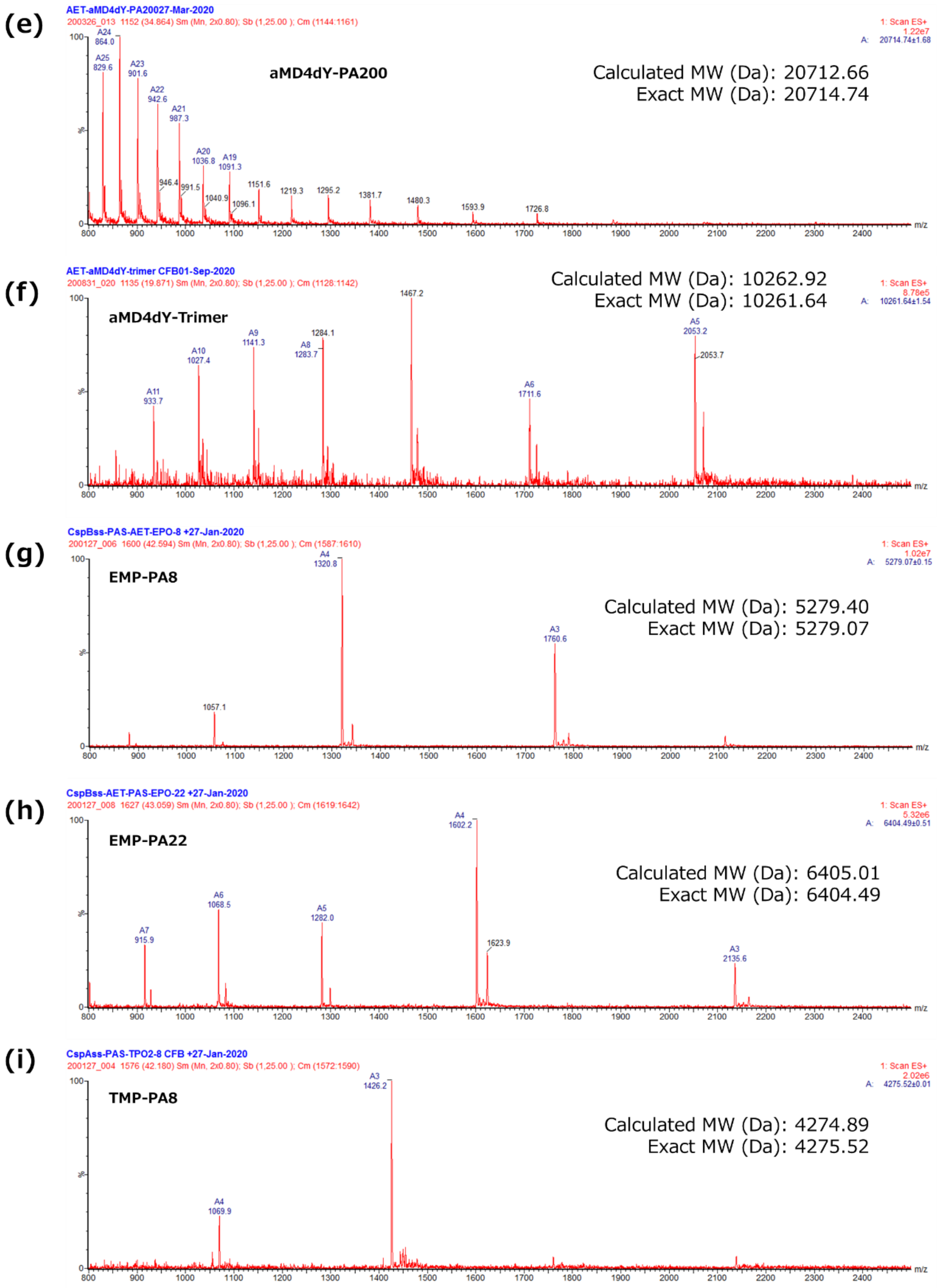

Supplementary Figure 2: MS analysis of supernatants. *Corynebacterium glutamicum* supernatants that secreted (a) aMD4dY-PA8, (b) aMD4dY-PA22, (c) aMD4dY-PA49, (d) aMD4dY-PA100, (e) aMD4dY-PA200, (f) aMD4dY-trimer, (g) EMP-PA8, (h) EMP-PA22, and (i) TMP-PA8 were analyzed by LC-MS before purification. Calculated (most abundant) MW and exact MW are shown.

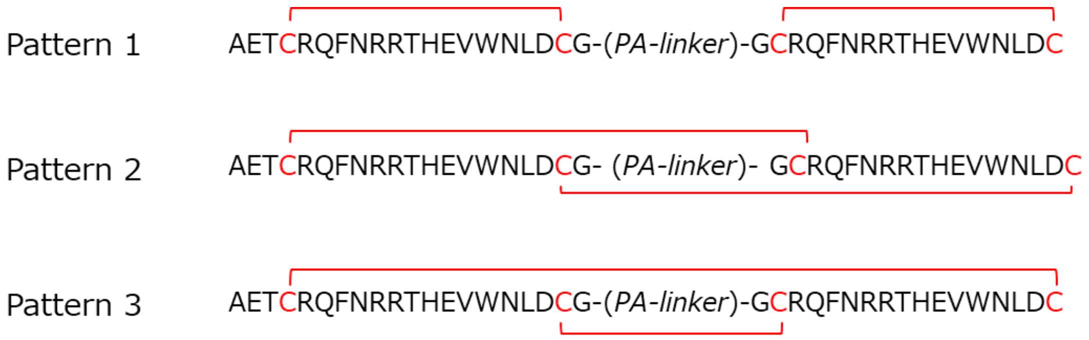

Supplementary Figure 3: Possible patterns of intracellular disulfide formation of aMD4dY-dimers. Three patterns of disulfide bonds (red lines) can be formed among four cysteine residues. The aMD4dY dimer with the designed disulfide pattern is shown as pattern 1, and the ones with the incorrect pattern as Patterns 2 and 3.

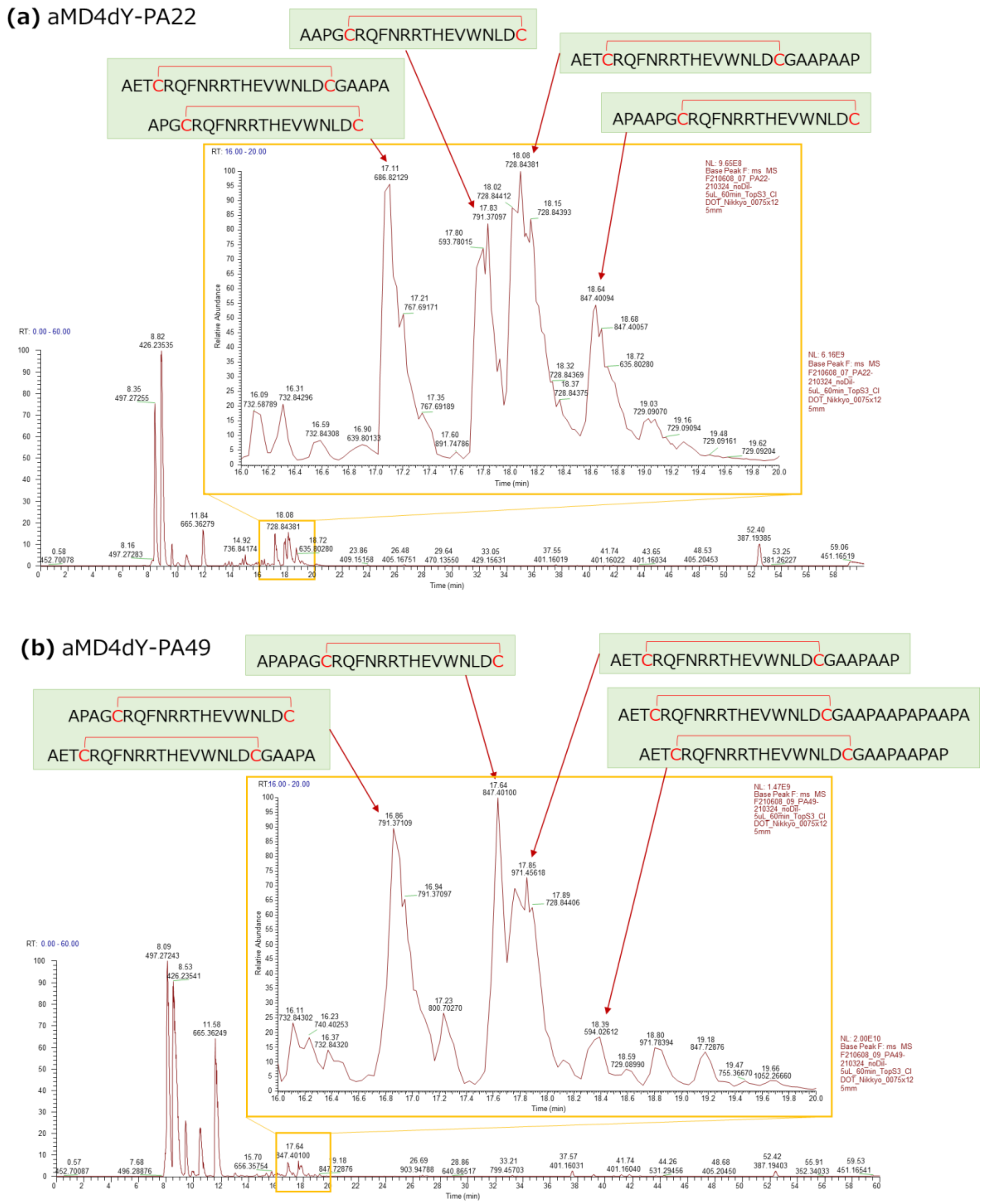

Supplementary Figure 4: Identification of disulfide bonds of STaMPtides using ProAlanase digestion followed by LC-MS/MS analysis. (a) aMD4dY-PA22 and (b) aMD4dY-PA49 were submitted for ProAlanase digestion without reductive alkylation and analyzed by LC-MS/MS. The base peak chromatogram of 0 to 60 min and magnified chromatogram of 16 to 20 min are shown and peaks of disulfide-containing

fragments are highlighted in green boxes. The peaks eluted from 8 to 12 min are derived from PA-linker, and the peaks eluted from 13 to 16.5 min are oxidated fragments.

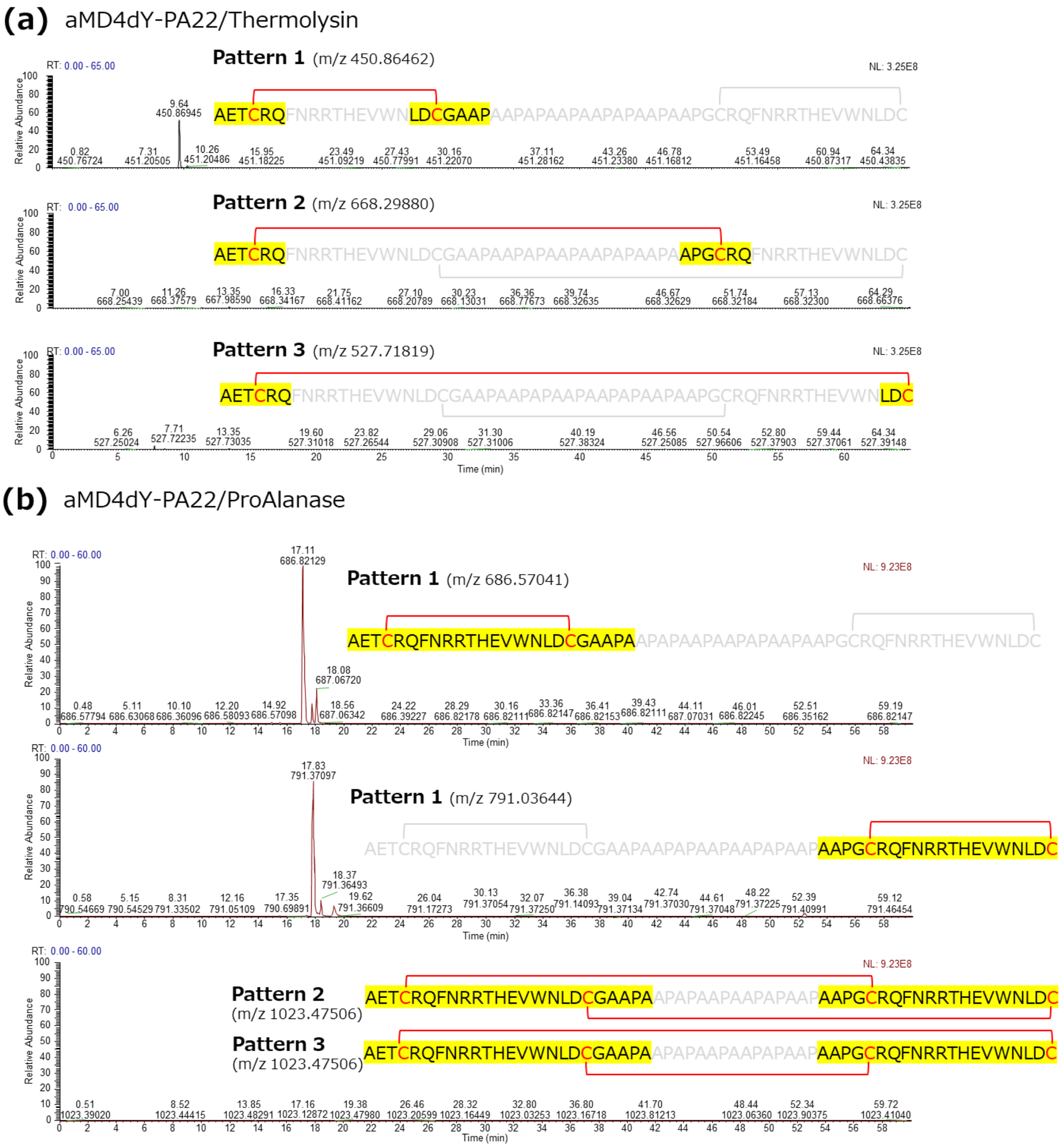

(a) aMD4dY-PA22/Thermolysin
Pattern 1 (m/z 450.86462)
AETCRQFNRRTHEVWNLDCGAAPAAPAPAAPAAPAPAAPAPAAPGCRQFNRRTHEVWNLDC
Pattern 2 (m/z 668.29880)
AETCRQFNRRTHEVWNLDCGAAPAAPAPAAPAAPAPAAPAPAAPGCRQFNRRTHEVWNLDC
Pattern 3 (m/z 527.71819)
AETCRQFNRRTHEVWNLDCGAAPAAPAPAAPAAPAPAAPAPAAPGCRQFNRRTHEVWNLDC
Time (min)
Relative Abundance
(b) aMD4dY-PA22/ProAlanase
Pattern 1 (m/z 686.57041)
AETCRQFNRRTHEVWNLDCGAAPAAPAPAAPAAPAPAAPAPAAPGCRQFNRRTHEVWNLDC
Pattern 1 (m/z 791.03644)
AETCRQFNRRTHEVWNLDCGAAPAAPAPAAPAAPAPAAPAPAAPGCRQFNRRTHEVWNLDC
Pattern 2 (m/z 1023.47506)
AETCRQFNRRTHEVWNLDCGAAPAAPAPAAPAAPAPAAPAAPGCRQFNRRTHEVWNLDC
Pattern 3 (m/z 1023.47506)
AETCRQFNRRTHEVWNLDCGAAPAAPAPAAPAAPAPAAPAAPGCRQFNRRTHEVWNLDC
Time (min)
Relative Abundance

(c) aMD4dY-PA49/ProAlanase

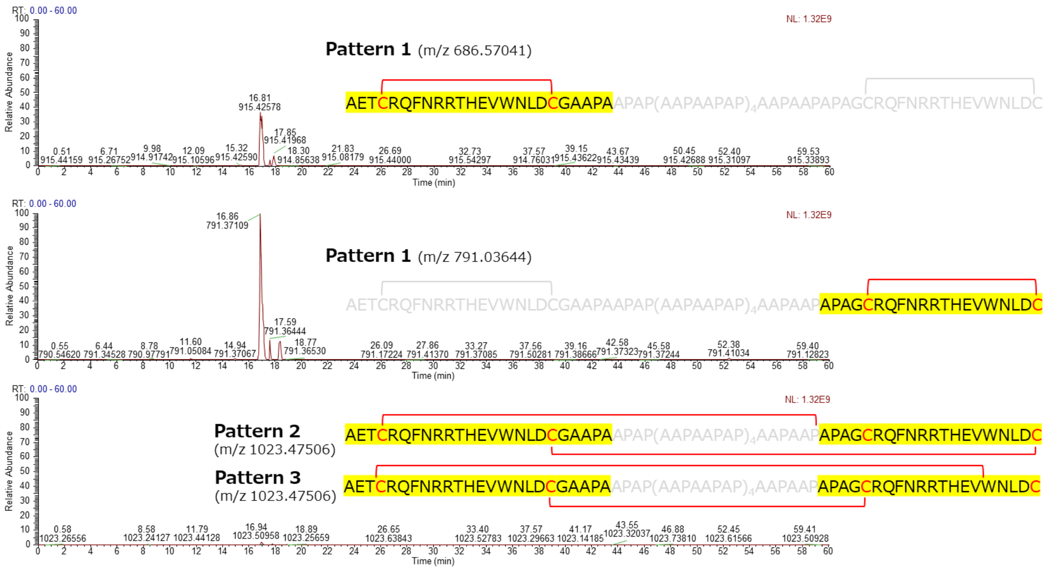

Supplementary Figure 5: The extracted ion chromatogram (XIC) of peptide fragments of (a) thermolysin-digested aMD4dY-PA22, (b) ProAlanase-digested aMD4dY-PA22 and (c) aMD4dY-PA49. Chromatograms of each peptide highlighted in yellow and with the indicated m/z are depicted. In each case, fragments derived from pattern 1 were detected, whereas fragments derived from patterns 2 and 3 were not.

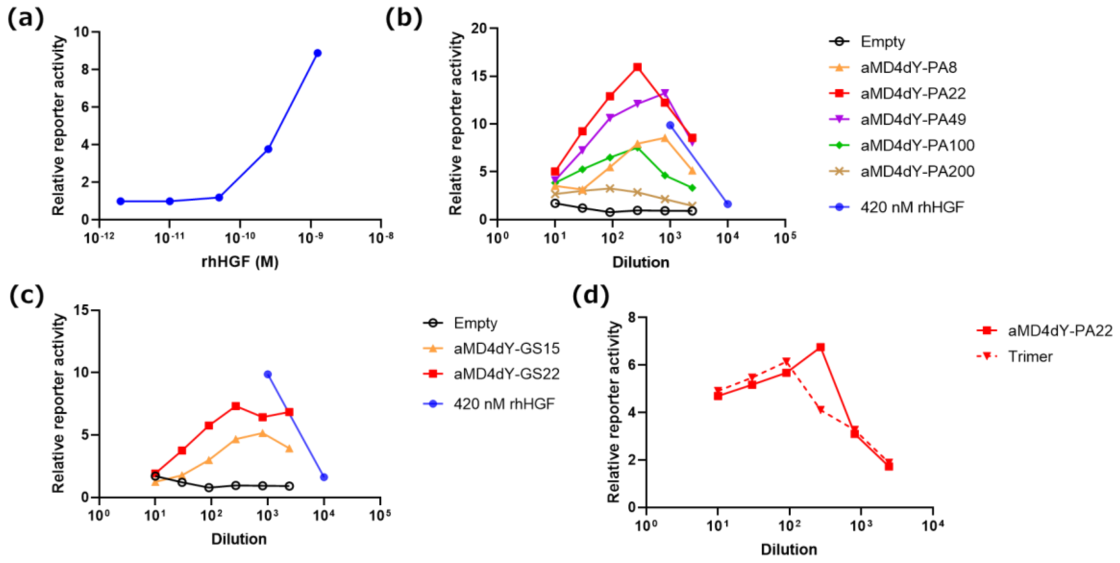

Supplementary Figure 6: Rapid activity-based screening of HGF mimetic STaMPtides with PA linkers by SRE reporter assay. (a) Dose-dependent response of SRE reporter by rhHGF stimulation. (b) Dose-dependent response of SRE reporter by Pro-Ala linker variants of HGF mimetic STaMPtides, aMD4dY-PA8 (orange triangles), aMD4dY-PA22 (red squares), aMD4dY-PA49 (purple inverted triangles), aMD4dY-PA100 (green rhombuses), and aMD4dY-PA200 (brown crosses). As controls, the diluted supernatant without STaMPtide secretion (empty, black open circle) and 420 nM rhHGF (blue circle) are also shown. (c) Dose-dependent response of SRE reporter by GS-linker variants of HGF mimetic STaMPtides, aMD4dY-GS15 (orange triangles) and aMD4dY-GS22 (red squares). The diluted supernatant without STaMPtide secretion (black open empty circle) and 420-nM rhHGF (blue circle) are also shown as controls. (d) Dose-dependent response of SRE reporter by HGF mimetic trimer STaMPtide (inverted triangles and broken line) and dimer STaMPtide (aMD4dY-PA22, red squares). All reporter activities were normalized by those without stimulation from the results of N=1.

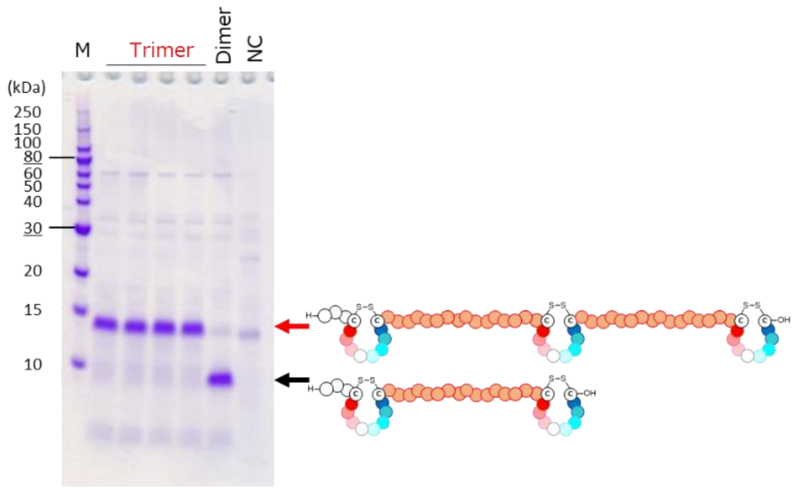

Supplementary Figure 7: SDS-PAGE analysis of secreted aMD4dY-trimer. For comparison, the dimer (aMD4dY-PA22) was analyzed. Red and black arrows indicate trimer and dimer STaMPtides, respectively. Red dots indicate STaMPtide-derived bands. M: Marker, NC: negative control supernatant (mock vector transfected).

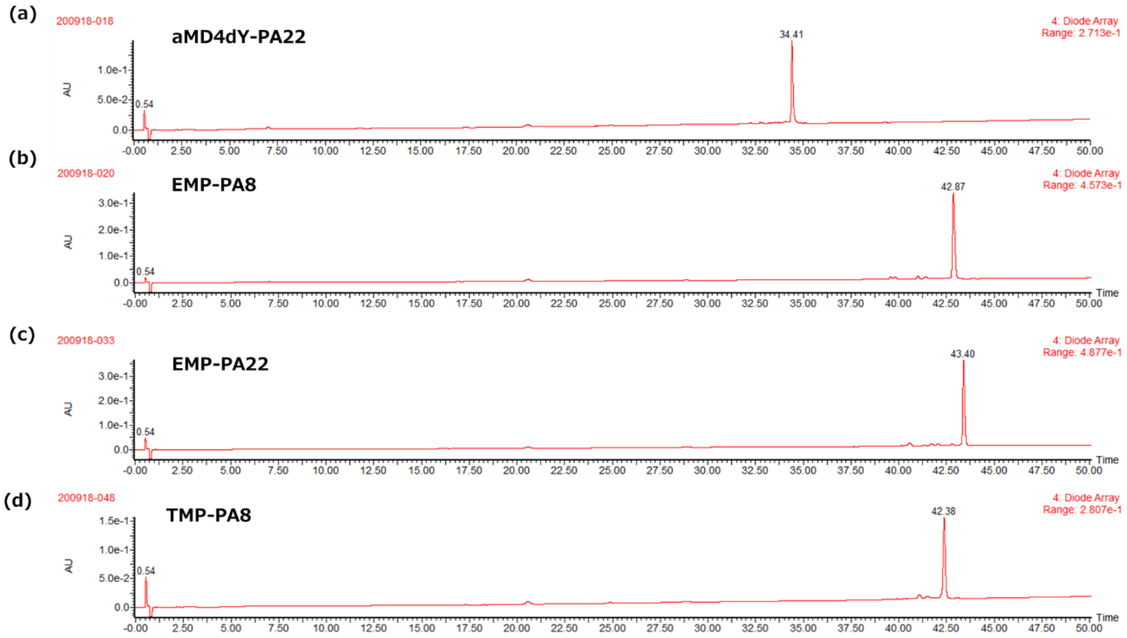

Supplementary Figure 8: Analytical LC measurement of purified STaMPtides. HPLC-purified (a) aMD4dY-PA22, (b) EMP-PA8, (c) EMP-PA22, and (d) TMP-PA8 were subjected to LC-MS analysis.

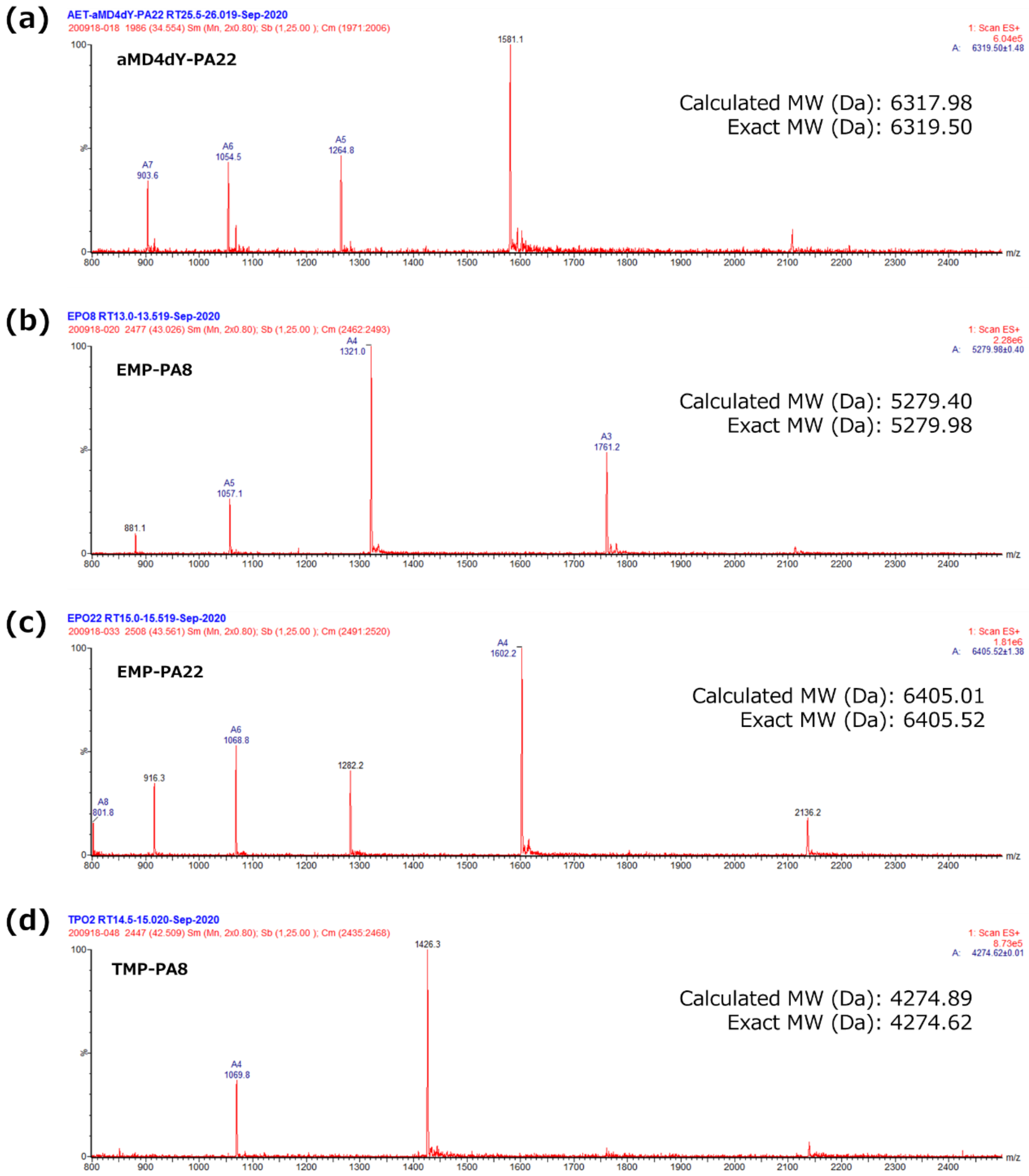

Supplementary Figure 9: MS analysis of purified STaMPtides. HPLC-purified (a) aMD4dY-PA22, (b) EMP-PA8, (c) EMP-PA22, and (d) TMP-PA8 were subjected to LC-MS analysis, and exact masses of main peaks shown in **Supplementary Fig. 8** are shown. Calculated (most abundant) MW and exact MW are shown.

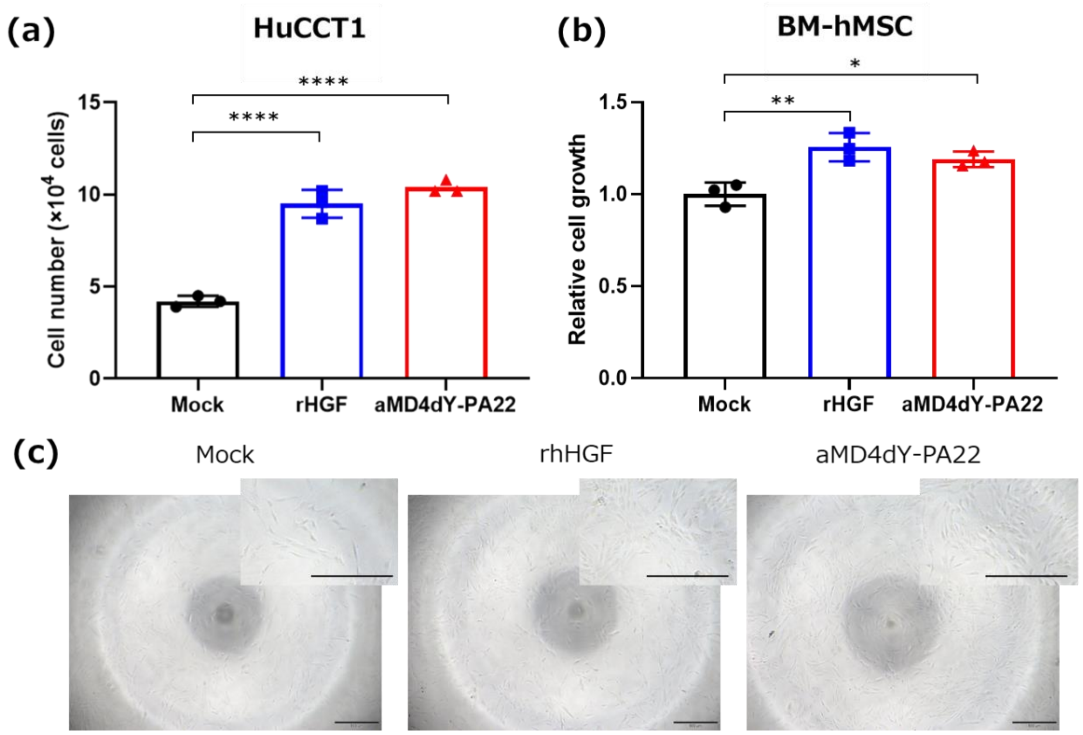

Supplementary Figure 10: Promotion of cell proliferation by aMD4dY-PA22 stimulation. (a) HuCCT1 cells were cultured in the absence (mock, black) or presence (blue) of 0.44 nM rhHGF or 6.3 nM aMD4dY-PA22 (red) for 5 days. Cell numbers were counted by an automated cell counter. The mean ± SD with individual values normalized by mock samples are shown from the results of triplicated experiments. ****$p < 0.0001$. (b) BM-hMSCs were cultured in the absence (mock, black) or presence (blue) of 1.3 nM rhHGF or 32 nM aMD4dY-PA22 (red) for 3 days. Cell numbers were counted by the WST-8 substrate. The mean ± SD with individual values normalized by mock samples are shown from the results of quadruplicated experiments. *$p < 0.05$ and **$p < 0.01$. (c) Morphology of BM-hMSCs at day 3. Scale bars = 500 µm.

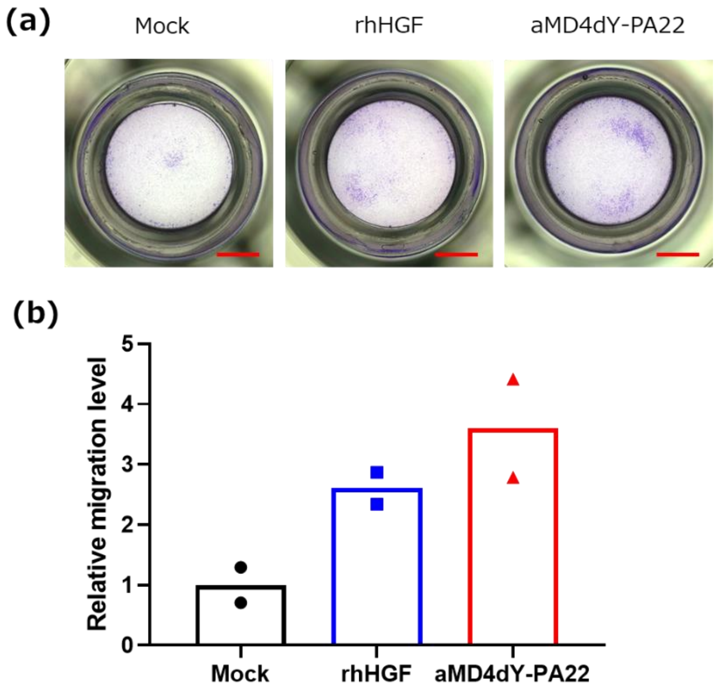

Supplementary Figure 11: Promotion of cell migration by aMD4dY-PA22 determined by Transwell assay. (a) Images of migrated cells on Transwell insert. HuCCT1 cells were cultured on Transwell inserts in the absence (mock) or presence of stimulation by 1.3 nM rhHGF or 32 nM aMD4dY-PA22 for 24 h. Cells on the basal side of Transwell inserts were fixed and stained with crystal violet. Scale bars = 2 mm. (b) Relative migration levels. Areas of stained HuCCT1 cells on Transwell inserts in the absence (mock, black) or presence (blue) of stimulation by 1.3 nM rhHGF or 32 nM aMD4dY-PA22 (red) for 24 h were quantified, Relative migration levels were standardized by those of the mock sample. The mean value and each data point in duplicated experiments are shown.

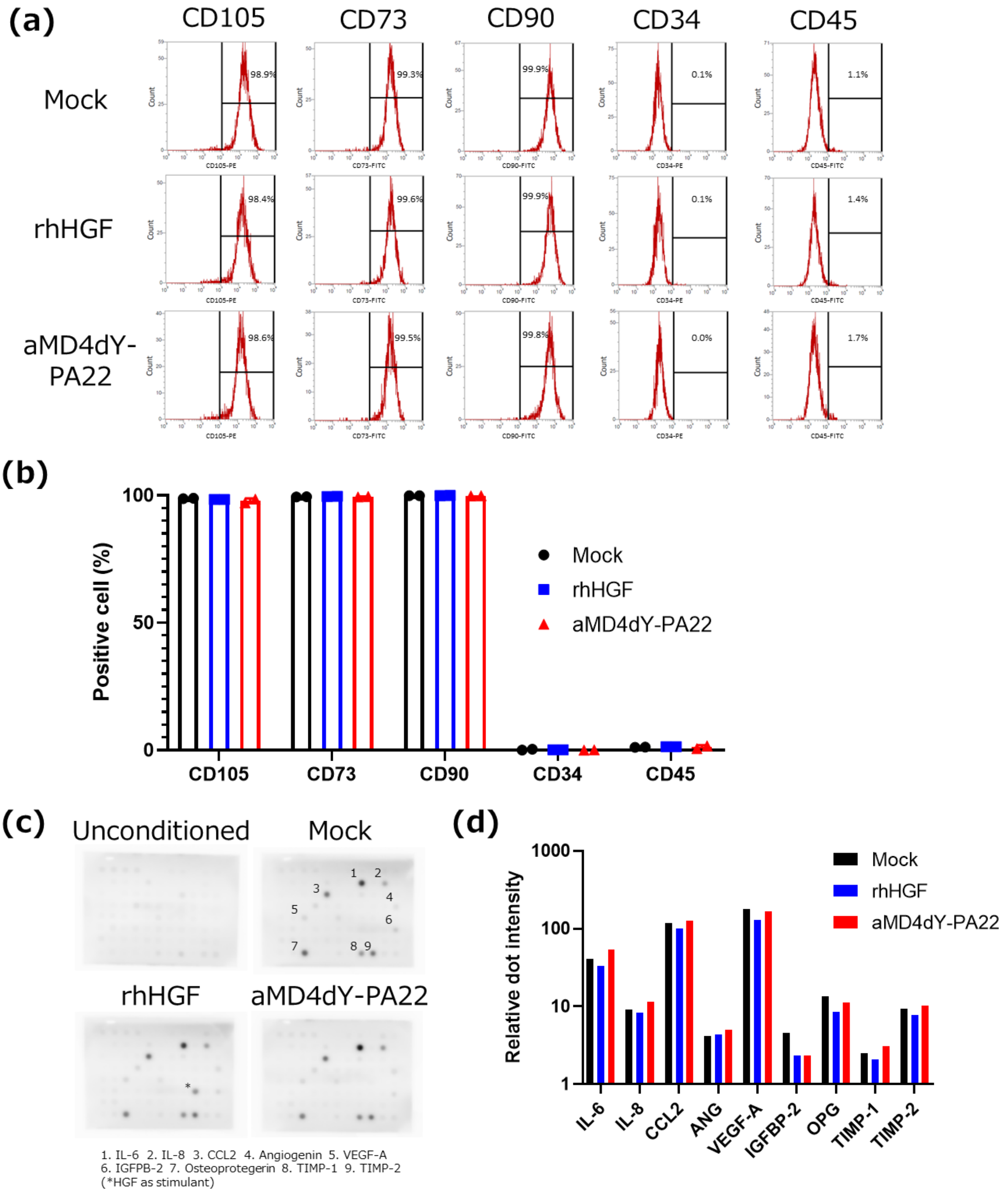

Supplementary Figure 12: Characterization of BM-hMSCs. (a) Flow cytometric analysis of cell surface marker expressions of BM-hMSCs cultured with or without 0.44 nM rhHGF or 6.3 nM aMD4dY-PA22 for 3 days. Representative histograms of positive markers CD105, CD73, and CD90 and negative markers CD34 and CD45 of each cell are shown. (b) Expression levels of each marker are shown as the mean values with each data point from the results of duplicated experiments. (c) Secreted cytokines in conditioned media of BM-hMSC unstimulated (mock) or stimulated by 0.44 nM rhHGF

or 6.3 nM aMD4dY-PA22 were analyzed using cytokine array. Unconditioned medium (D-MEM/F12 + 10% FBS) was analyzed as the negative control. Cytokines whose content was increased by conditioning were marked. Asterisk indicates detection of rhHGF as stimulant. (d) Quantification of relative intensities of dots selected in **Supplementary Fig. 12(b).** Each dot intensities of the conditioned medium were normalized by those of unconditioned medium.

(a)

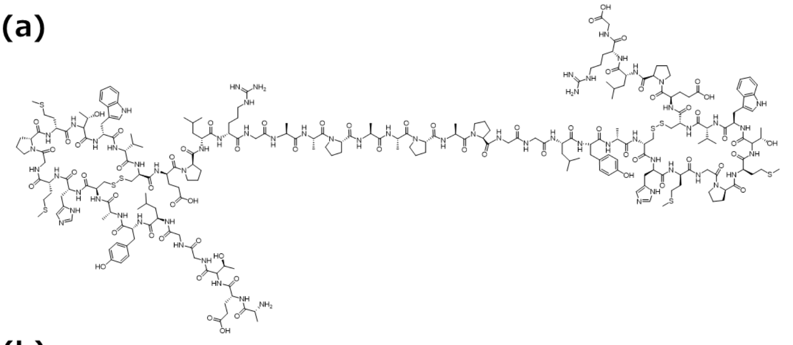

(b)

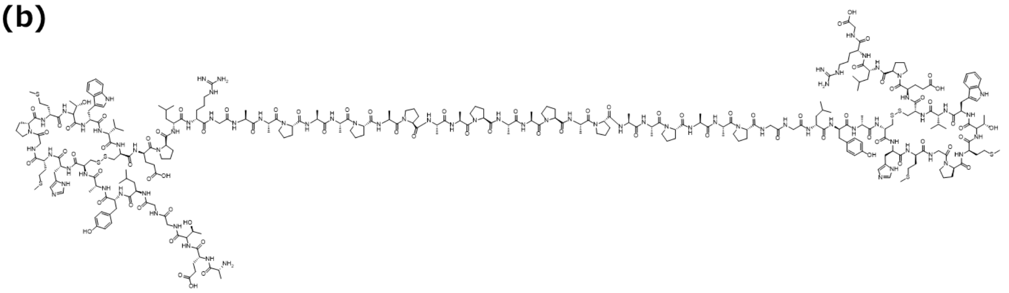

(c)

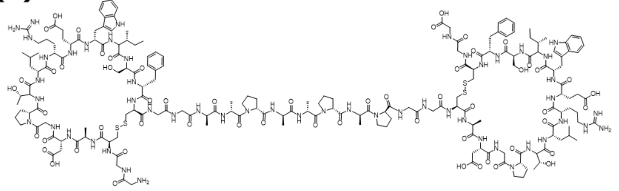

Supplementary Figure 13: Chemical structures of (a) EMP-PA8, (b) EMP-PA22, and (c) TMP-PA8.

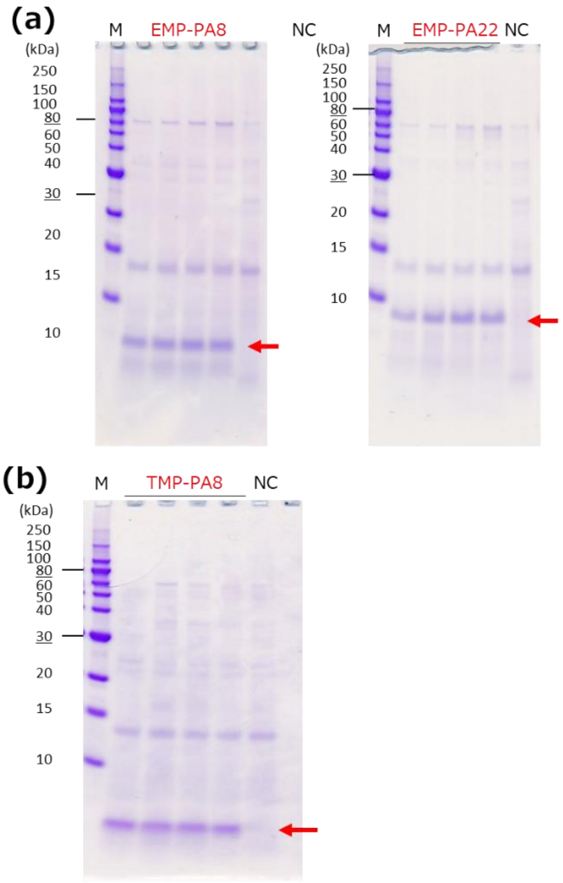

Supplementary Figure 14: SDS-PAGE analysis of secreted (a) EPO mimetic (EMP-PA8 and EMP-PA22) and (b) TPO mimetic (TMP-PA8) STaMPtides. Red arrows indicate STaMPtide-derived bands. M: Marker, NC: negative control supernatant (mock vector transfected).

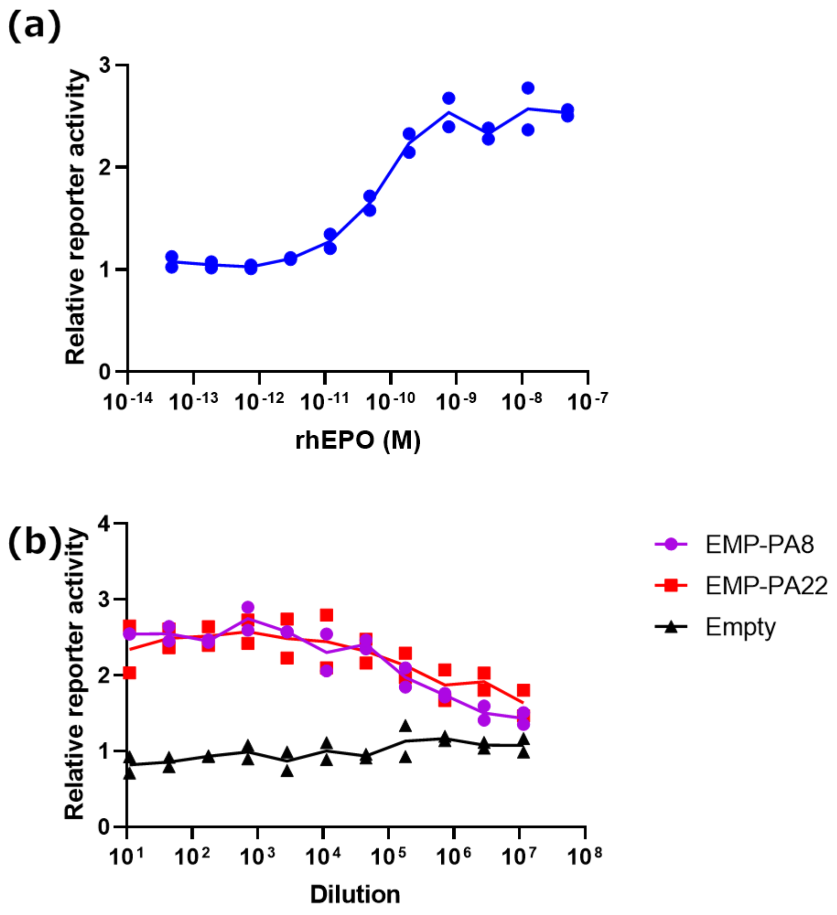

Supplementary Figure 15: Evaluation of EPO mimetic STaMPtide-secreted supernatants. (a) Dose-dependent activity of EPOR-JAK2 functional reporter by rhEPO stimulation. (b) Dose-dependent response of EPO mimetic STaMPtides. Reporter activities of stimulation by diluted supernatants with EMP-PA8 (purple circles) and EMP-PA22 (red squares) or the control supernatant (mock-vector-transfected, black triangles) were quantified. The mean and individual values are shown from the results of duplicate experiments.

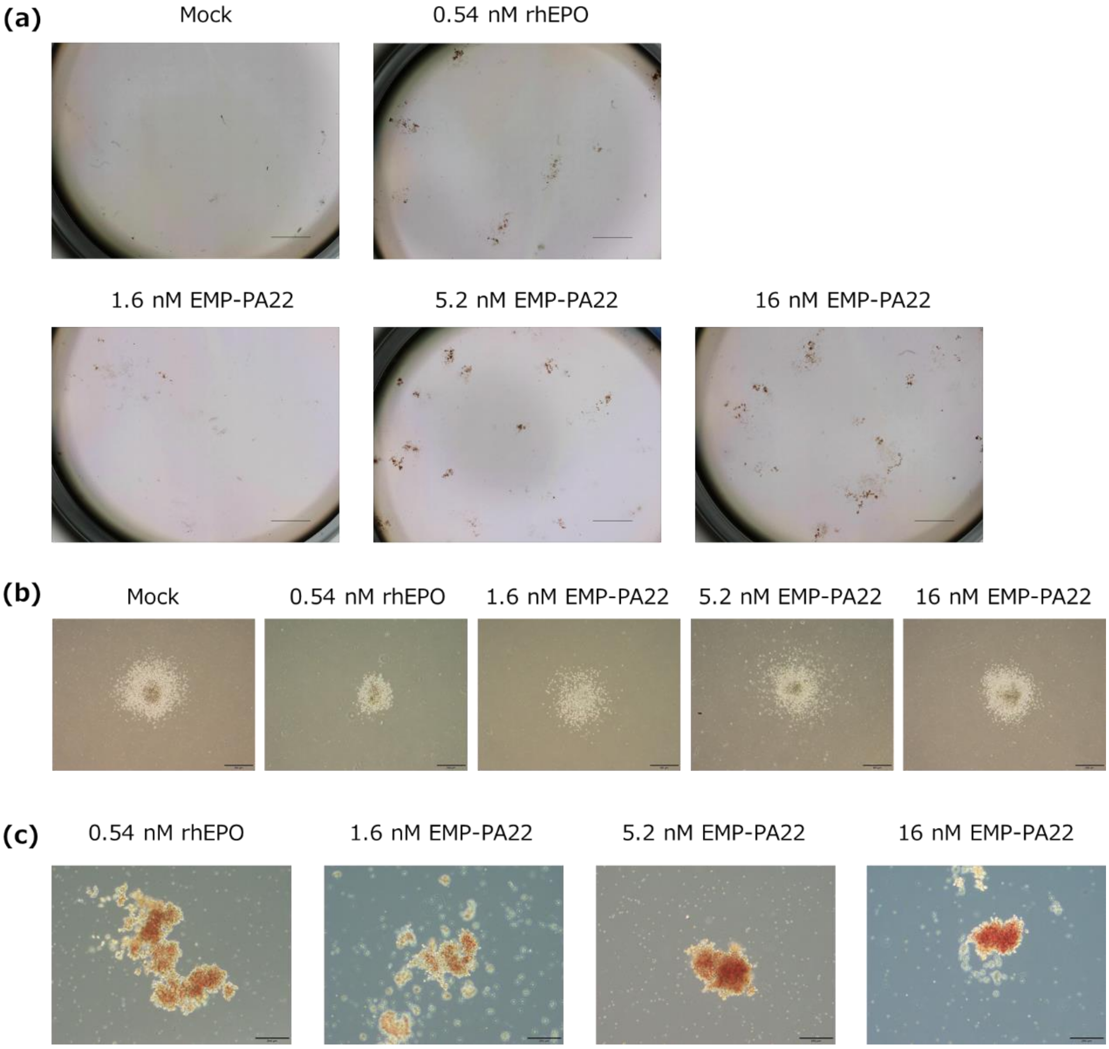

Supplementary Figure 16: Morphologies of erythroid colonies formed by hPBMCs. (a) Images of culture wells of colony-forming cell assay. Scale bars = 4 mm. (b) Images of representative myeloid colonies at day 13 in the absence or presence of stimulations. Scale bars = 500 μm. (c) Images of representative erythroid colonies at day 13 in the presence of stimulations. No colony was formed without rhEPO or EPO mimetic STaMPtide stimulation. Scale bars = 200 μm.

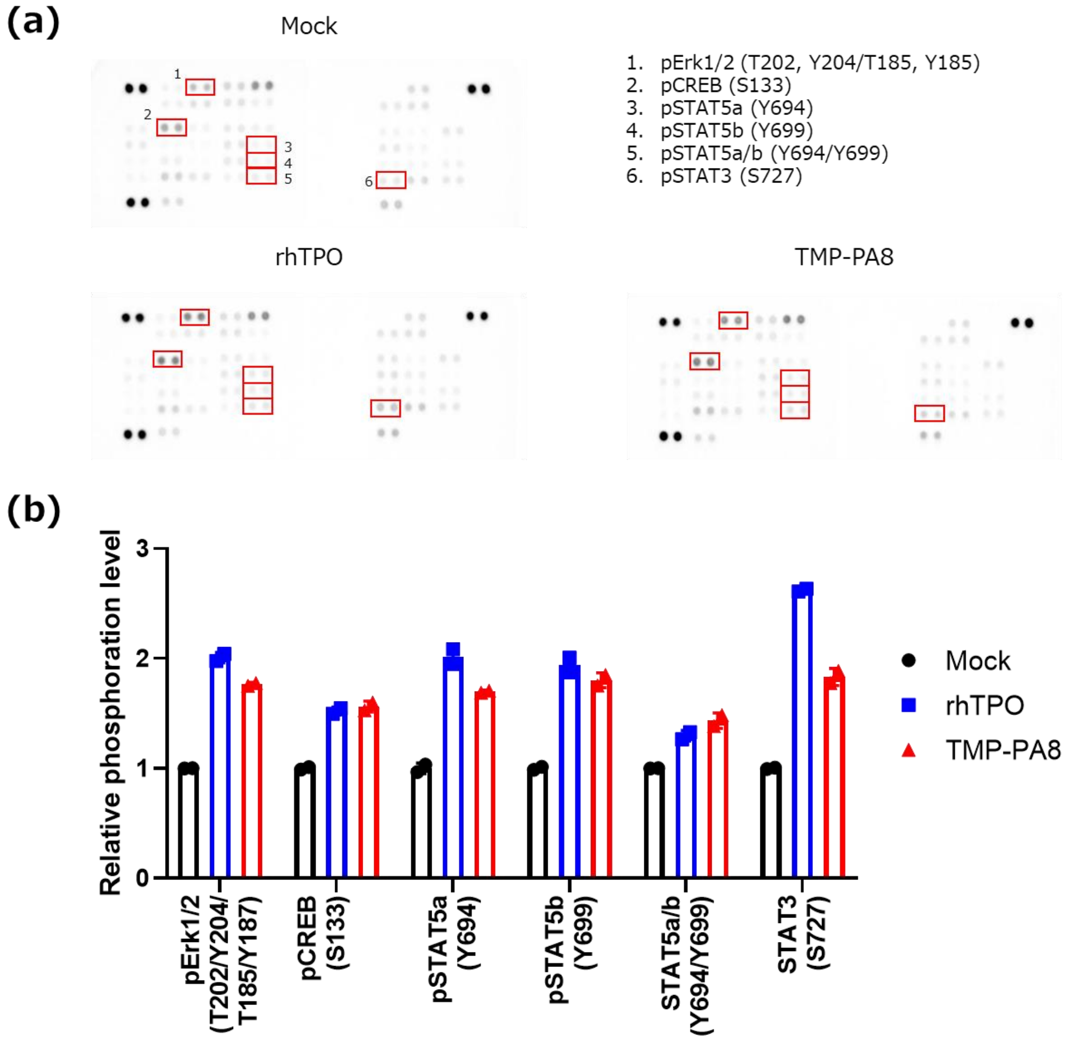

Supplementary Figure 17: Signal propensity of TMP-PA8. **a** Phosphokinase array analysis. Lysates of HEL cells stimulated by 5.4 nM rhTPO or 1:1000 diluted TMP-PA8 supernatant for 20 min were analyzed by a phosphokinase array. Positions of kinases whose phosphorylation was promoted are indicated by red boxes. (b) Quantification of relative phosphorylation levels of kinases quantified from the dot intensity of the arrays and normalized by those of unstimulated cells.

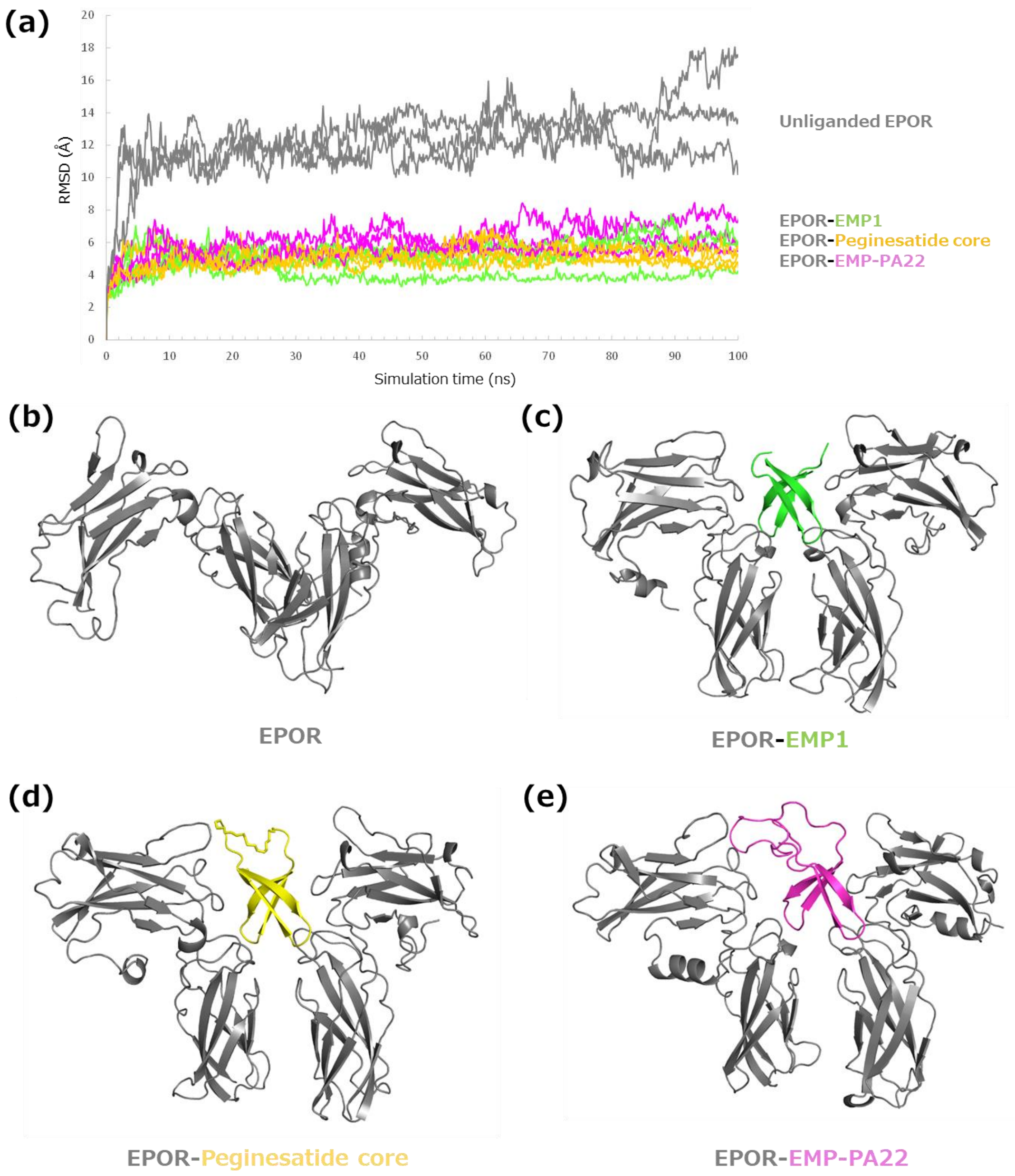
(a)
RMSD (Å)
Simulation time (ns)
0
2
4
6
8
10
12
14
16
18
20
0
10
20
30
40
50
60
70
80
90
100
Unliganded EPOR
EPOR-EMP1
EPOR-Peginesatide core
EPOR-EMP-PA22
(b)
EPOR
(c)
EPOR-EMP1
(d)
EPOR-Peginesatide core
(e)
EPOR-EMP-PA22

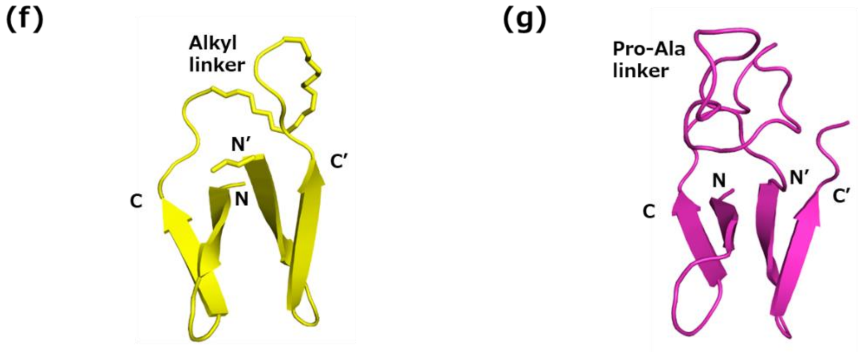

Supplementary Figure 18: Structural prediction of EPOR–ligand complexes. Complexes of EPOR ectodomain and EMP1, peginesatide core, or EMP-PA22 were predicted by 100 ns MD simulation from the previously reported EPOR-EMP1 crystal structure (PDB: 1EBP). (a) RMSD plots of EPOR–ligand models during 100 ns MD simulation. Results in triplicated simulations are shown. (b–e) Representative predicted structures of (b) 2 molecules of EPOR without ligand, (c) 2:2 EPOR–EMP1 complex, (d) 2:1 EPOR–peginesatide core complex, and (e) 2:1 EPOR–EMP-PA22 complex at a structurally stable period (30–100 nm). (f and g) Magnified images of predicted structures of linked termini of (f) peginesatide core and (g) EMP-PA22 in complex with EPOR. N-termini (N, N') and C-termini (C, C') of EPOR-binding peptide domains of each ligand are depicted.
