# Supplementary material for: Single-chain tandem macrocyclic peptides as a scaffold for growth factor and cytokine mimetics
Source: Commun Biol. 2022 Jan 14;5:56. doi: 10.1038/s42003-022-03015-6 (PMC8760323; doi:10.1038/s42003-022-03015-6)
Supplement: Supplementary file 3 — Description of Additional Supplementary Files [file 42003_2022_3015_MOESM3_ESM.pdf]

# Description of Additional Supplementary Files

**File name:** Supplementary Data 1
**Description:** Source data for Figures 4 and 5 and Supplementary Figures 6, 10, 11, 12, 15, and 17. Detailed source data underlying the means shown in Supplementary Table 1 are also included.
