# Supplementary material for: Single-chain tandem macrocyclic peptides as a scaffold for growth factor and cytokine mimetics
Source: Commun Biol. 2022 Jan 14;5:56. doi: 10.1038/s42003-022-03015-6 (PMC8760323; doi:10.1038/s42003-022-03015-6)
Supplement: Supplementary file 5 — Reporting Summary [file 42003_2022_3015_MOESM5_ESM.pdf]

nature portfolio

Corresponding author(s): Kenichiro Ito

Last updated by author(s): Dec 24, 2021

Reporting Summary

Nature Portfolio wishes to improve the reproducibility of the work that we publish. This form provides structure for consistency and transparency in reporting. For further information on Nature Portfolio policies, see our Editorial Policies and the Editorial Policy Checklist.

Statistics

For all statistical analyses, confirm that the following items are present in the figure legend, table legend, main text, or Methods section.

n/a	Confirmed	
☐	☒	The exact sample size (*n*) for each experimental group/condition, given as a discrete number and unit of measurement
☐	☒	A statement on whether measurements were taken from distinct samples or whether the same sample was measured repeatedly
☐	☒	The statistical test(s) used AND whether they are one- or two-sided<br>*Only common tests should be described solely by name; describe more complex techniques in the Methods section.*
☒	☐	A description of all covariates tested
☒	☐	A description of any assumptions or corrections, such as tests of normality and adjustment for multiple comparisons
☐	☒	A full description of the statistical parameters including central tendency (e.g. means) or other basic estimates (e.g. regression coefficient) AND variation (e.g. standard deviation) or associated estimates of uncertainty (e.g. confidence intervals)
☐	☒	For null hypothesis testing, the test statistic (e.g. *F*, *t*, *r*) with confidence intervals, effect sizes, degrees of freedom and *P* value noted<br>*Give P values as exact values whenever suitable.*
☒	☐	For Bayesian analysis, information on the choice of priors and Markov chain Monte Carlo settings
☒	☐	For hierarchical and complex designs, identification of the appropriate level for tests and full reporting of outcomes
☒	☐	Estimates of effect sizes (e.g. Cohen's *d*, Pearson's *r*), indicating how they were calculated

Our web collection on statistics for biologists contains articles on many of the points above.

Software and code

Policy information about availability of computer code

Data collection	MD simulation was performed using the Bioluminate software suite version 2020-3.
Data analysis	Data were analyzed using GraphPad Prism 8, Biacore T200, ChemDraw 19.0, BZ-H3, MassLynx, XCalibur. Attune NxT Software 2.6.580.1.

For manuscripts utilizing custom algorithms or software that are central to the research but not yet described in published literature, software must be made available to editors and reviewers. We strongly encourage code deposition in a community repository (e.g. GitHub). See the Nature Portfolio guidelines for submitting code & software for further information.

Data

Policy information about availability of data

All manuscripts must include a data availability statement. This statement should provide the following information, where applicable:

- Accession codes, unique identifiers, or web links for publicly available datasets
- A description of any restrictions on data availability
- For clinical datasets or third party data, please ensure that the statement adheres to our policy

All the data supporting findings of the research are available in Figures 1-5, Supplementary Figures 1-18 and Supplementary Tables 1-3. Source data are available in Supplementary Data 1 as source data. Data and detailed expression methods including full plasmid sequences are able to be provided by the corresponding author upon reasonable requests.

Field-specific reporting

Please select the one below that is the best fit for your research. If you are not sure, read the appropriate sections before making your selection.

☒ Life sciences ☐ Behavioural & social sciences ☐ Ecological, evolutionary & environmental sciences

For a reference copy of the document with all sections, see nature.com/documents/nr-reporting-summary-flat.pdf

Life sciences study design

All studies must disclose on these points even when the disclosure is negative.

Sample size	For data requiring statistical analysis, a sample size of three or more was selected (Figs. 3a, 3b, 3d, 4b, Supplementary Figs. 10a, 10b, Supplementary Table 1). This criterion is judged to be sufficient to show the difference between the control and test samples due to its small variability.
Data exclusions	No data were excluded.
Replication	Experiments were replicated to confirm reproducibility.
Randomization	No randomization is required for this study. The same cell source is used for in the same experiment.
Blinding	No blinding was applied in this study.

Reporting for specific materials, systems and methods

We require information from authors about some types of materials, experimental systems and methods used in many studies. Here, indicate whether each material, system or method listed is relevant to your study. If you are not sure if a list item applies to your research, read the appropriate section before selecting a response.

Materials & experimental systems

n/a	Involved in the study	
☐	☒	Antibodies
☐	☒	Eukaryotic cell lines
☒	☐	Palaeontology and archaeology
☒	☐	Animals and other organisms
☒	☐	Human research participants
☒	☐	Clinical data
☒	☐	Dual use research of concern

Methods

n/a	Involved in the study	
☒	☐	ChIP-seq
☐	☒	Flow cytometry
☒	☐	MRI-based neuroimaging

Antibodies

Antibodies used	Antibodies for flow cytometry: FITC anti-human CD90 (Biolegend 328107), PE anti-human CD105 (Biolegend 323205), PE anti-human CD34 (Biolegend 343605), FITC anti-human CD45 (Biolegend 304005), FITC-anti CD73(Biolegend 344016), PE mouse IgG1 k Isotype (Biolegend 400139), FITC mouse IgG1 k Isotype (Biolegend 400110)<br>Antibodies for cell-based ELISA: Phospho-Met (Tyr1234/1235) (D26) XP Rabbit mAb (Cell Signaling Technology 3077), Anti-mouse IgG, HRP-linked Antibody (Cell Signaling Technology 7076)
Validation	All the antibodies for flow cytometry are compatible with flow cytometry as stated on the manufacturer's website, "Each lot of this antibody is quality control tested by immunofluorescent staining with flow cytometric analysis."<br>Antibodies for cell-based ELISA are selected following to previous reports; Nat. Commun. 6, 6373 (2015) and Int. J. Mol. Sci. 19, 3141 (2018).

Eukaryotic cell lines

Policy information about cell lines

Cell line source(s)	HuCCT1, HEL (JCRB), HEK293 (ATCC), BM-hMSCs and hPBMCs (Lonza) are used in the study.
Authentication	Cells were not authenticated.
Mycoplasma contamination	Cells were not tested for mycoplasma contamination.

Commonly misidentified lines (See ICLAC register)	No commonly misidentified cell lines were used.

Flow Cytometry

Plots

Confirm that:

☒ The axis labels state the marker and fluorochrome used (e.g. CD4-FITC).

☒ The axis scales are clearly visible. Include numbers along axes only for bottom left plot of group (a 'group' is an analysis of identical markers).

☒ All plots are contour plots with outliers or pseudocolor plots.

☒ A numerical value for number of cells or percentage (with statistics) is provided.

Methodology

Sample preparation	Harvested cells were blocked by PBS supplemented with Human TruStain FcX (Biolegend) for 10 min on ice. FITC anti-human CD90 (Biolegend), PE anti-human CD105 (Biolegend), PE anti-human CD34 (Biolegend), FITC anti-human CD45 (Biolegend) or FITC-anti CD73 (Biolegend) diluted for 10 times by cell stain buffer (PBS supplemented with 1 % FBS) were added to cells and incubated for 1 h on ice. For isotype controls, 1:10 diluted PE mouse IgG1 k Isotype (Biolegend) and FITC mouse IgG1 k Isotype (Biolegend) were used. After washing two times by cell stain buffer, cells were analyzed by Attune NxT flow cytometer (Thermo Fisher Scientific).
Instrument	Attune NxT (Thermo Fisher Scientific)
Software	Attune NxT Software 2.6.580.1
Cell population abundance	Abundance of the relevant cell populations were determined by positive/negative marker ratio of flow cytometry analysis.
Gating strategy	Cell debris was excluded by low FSC/SSC gating. Isotype controls of mouse IgGk were used to indicate boundaries of positive/negative staining.

☒ Tick this box to confirm that a figure exemplifying the gating strategy is provided in the Supplementary Information.
